# The Utility of Thoracic Ultrasound in Patients with Acute Eosinophilic Pneumonia

**DOI:** 10.1371/journal.pone.0124370

**Published:** 2015-04-20

**Authors:** Hee Yoon, Se Jin Kim, Kang Kim, Ji Eun Lee, Byung Woo Jhun

**Affiliations:** 1 Department of emergency medicine, Samsung Medical Center, Sungkyunkwan University School of Medicine, Seoul, South Korea; 2 Division of Pulmonary and Critical Care Medicine, Department of Medicine, The Armed Forces Capital Hospital, Seong-nam, South Korea; Clinica Universidad de Navarra, SPAIN

## Abstract

Thoracic ultrasound (TUS) is an easy-to-use imaging modality that aids physicians in the differential diagnosis of respiratory diseases. However, no data exist on the TUS findings of acute eosinophilic pneumonia (AEP) or their clinical utility in patients with AEP. Thus, we performed an observational study on TUS findings and their clinical utility for follow-up in patients with AEP. We prospectively screened patients who visited the emergency department for acute respiratory symptoms at the Armed Forces Capital Hospital in South Korea between February 2014 and July 2014. Of them, patients suspected to have AEP underwent an etiological investigation, including flexible bronchoscopy with bronchoalveolar lavage and TUS, and we evaluated TUS findings and serial changes on TUS during the treatment course compared with those from chest radiographs. In total, 22 patients with AEP were identified. The TUS examinations reveled that all patients exhibited multiple diffuse bilateral B-lines and lung sliding, with (*n* = 5) or without pleural effusion, which was consistent with alveolar-interstitial syndrome. B-line numbers fell during the course of treatment, as the lines became thinner and fainter. A-lines were evident in 19 patients on day 7 of hospitalization, when B-lines had disappeared in 13 patients, and all pleural effusion had resolved. All patients exhibited complete ultrasonic resolution by day 14, along with clinicoradiological improvement. Chest radiographs of five patients taken on day 7 seemed to show complete resolution, but several abnormal B-lines were evident on TUS performed the same day. As a result, our data show common TUS findings of AEP and suggest that AEP may be included as a differential diagnosis when multiple diffuse bilateral B-lines with preserved lung sliding are identified on a TUS examination in patients with acute symptoms, and that TUS is a useful modality for evaluating the treatment response in patients with AEP.

## Introduction

Thoracic ultrasound (TUS) is a non-invasive and readily available imaging modality that allows physicians to evaluate the causes of respiratory failure and thoracic disease at the point of care, without exposure to radiation [1]. Studies on the clinical applicability of TUS have consistently indicated that the technique is diagnostically satisfactory when used to evaluate acute respiratory failure. The data are comparable in quality to those yielded by chest radiography, particularly when patients have been admitted to an intensive care unit (ICU) or emergency department [2–4], and help distinguish respiratory diseases, including pneumonia, pulmonary edema, pleural effusion, pneumothorax, alveolar-interstitial syndrome, and pulmonary embolism [5–7]. The recent establishment of a systematic TUS evaluation protocol has encouraged efforts to increase the applicability of TUS in various medical situations [8].

Our institute has the highest referral rate in South Korea and annually recruits >70 cases of acute eosinophilic pneumonia (AEP) [9–12]. AEP is an uncommon inflammatory lung disease of unknown etiology, characterized by acute respiratory symptoms, diffuse radiographic pulmonary infiltrates, and infiltration of eosinophils into the lung, in the absence of a known cause for the pulmonary eosinophilia [13]. As some AEP cases are severe, we seek favorable outcomes by encouraging early suspicion of the disease because the clinical response to corticosteroids is quite dramatic, even in fatal cases.

However, no data are available on typical TUS findings of AEP or the clinical utility of follow-up in patients with AEP. Thus, in the present study, we investigated common TUS findings of AEP at initial presentation and assessed serial changes in TUS findings during the treatment course compared with those of chest radiography to evaluate the clinical utility for follow-up in patients with AEP.

## Materials and Methods

### Study subjects

We prospectively screened patients who visited the emergency department for acute respiratory symptoms at the Armed Forces Capital Hospital (874-bed military referral hospital) in South Korea between February 2014 and July 2014. All screened patients underwent a conventional diagnostic work-up, including history-taking, physical examinations, chest radiography, and laboratory tests. Of all patients, those suspected of AEP, who had recently altered their smoking habit; who presented with acute-onset respiratory symptoms; who exhibited diffuse lung infiltration on chest radiography; or who had peripheral eosinophilia [9] were subjected to a thorough disease etiology investigation using TUS, chest computed tomography (CT), and flexible diagnostic bronchoscopy with bronchoalveolar lavage (BAL).

A definitive AEP diagnosis [9] was based on fulfilling criteria modified from those proposed by Philit *et al*. [13]: 1) acute onset of febrile respiratory manifestations <1 month in duration; 2) bilateral diffuse infiltrate evident on chest radiography; 3) >25% eosinophils in BAL or eosinophilic pneumonia evident on lung biopsy; and 4) absence of any known cause of pulmonary eosinophilia, including drug abuse, toxin exposure, or infection. Patients confirmed to have AEP and who received TUS were included in the analysis, and the TUS findings were evaluated. All diagnostic procedures, including TUS and flexible bronchoscopy with BAL were performed after written informed consent was obtained. This study was approved by the Armed Forces Capital Hospital Institutional Review Board, which allowed us to review and publish data from the patient’s records (approval no. AFMC-14-IRB-030).

### Thoracic ultrasound examination

TUS (SequoiaC512 Acuson; Siemens, Munich, Germany) was usually performed with a linear array transducer (5–8 MHz), but a convex transducer (1–4 MHz) was employed occasionally. Each hemithorax was divided into three areas: anterior, lateral, and posterior. The anterior region ran from the parasternal to the anterior axillary line, the lateral region from the anterior axillary line to the posterior axillary line, and the posterior region from the posterior axillary line to the paravertebral line. Each of these three areas was divided into upper and lower zones. Thus, TUS findings were recorded for 12 distinct regions. Patients were anterolaterally examined while supine and examined posteriorly while seated. Ultrasound images, including both normal (lung sliding and A-lines) and abnormal (multiple diffuse B-lines, consolidation, and effusion) findings, were recorded.

TUS features were recorded as indicated previously and as follows [8, 14]: *Lung sliding*: depiction of a regular rhythmic movement synchronized with respiration that occurred between the parietal and visceral pleura that were either in direct apposition or separated by a thin layer of intrapleural fluid; *A-lines*: repetitive horizontal artifacts along the pleural line caused by sub-pleural air; *B-lines*: discrete laser-like vertical hyperechoic reverberation artifacts that arose from the pleural line (previously described as comet tails), extended to the bottom of the screen without fading, and moved synchronously with lung sliding. A positive region was defined by the presence of three or more B-lines in a longitudinal plane between two ribs; *consolidation*: a subpleural echo-poor region or one with tissue-like echotexture; and *pleural effusion*: a space (usually anechoic) between the parietal and visceral pleura with respiratory movement of the lung within the effusion (sinusoid sign). We simultaneously evaluated heart function using a microconvex transducer (1–4 MHz) when abnormal B-lines were observed on TUS to exclude cardiogenic pulmonary edema. TUS was usually performed before diagnostic flexible bronchoscopy with BAL, and follow-up was performed at 7-day intervals, but more frequently in some cases, at the discretion of the attending physician.

### Patient management and data collection

All patients diagnosed with AEP were treated with corticosteroids using a protocol described previously [9], according to the presence of respiratory failure (partial pressure of arterial oxygen [PaO_2_]/fraction of inspired oxygen ratio [FiO_2_] ratio ≤300 mmHg) and/or tachypnea (respiration rate >30 breaths/min). If a patient exhibited respiratory failure, 60-mg methylprednisolone was administered intravenously every 6 h for 3 days. If no respiratory failure was evident, 30-mg oral prednisolone was administered twice daily for 3 days. Corticosteroids were tapered over 2 weeks in all patients.

The following data were collected prospectively. Baseline characteristics, laboratory test data, including white blood cell count, serum levels of C-reactive protein and N-terminal prohormone brain natriuretic peptide, BAL fluid, radiographs, chest CT findings, and TUS. Serial changes in the TUS findings were collected according to clinical, laboratory, and radiological responses during treatment. The final follow-up data were obtained on July 20, 2014.

## Results

### Clinical characteristics

In total, 30 consecutive patients suspected of AEP were admitted to our emergency department during the study period. Of them, 22 were confirmed to have AEP and received TUS. The clinical characteristics of the 22 patients are shown in Table 1. Median patient age was 20 years, and all were males and current smokers. All patients presented with acute respiratory or systemic symptoms, including cough (*n* = 21), sputum (*n* = 8), fever (*n* = 22), or dyspnea (*n* = 21).

**Table 1:** Clinical characteristics of study patients.

Characteristic	
Patients	22 (100)
Age (years)	20 (20–21)
Gender (male)	22 (100)
Body mass index (kg/m^2^)	23.6 (21.4–25.0)
Current smoker	22 (100)
Acute presenting symptoms	
Cough	21 (96)
Sputum	8 (36)
Fever	22 (100)
Dyspnea (> MMRC scale II)	21 (96)
Laboratory findings	
White blood cell count (/μL)	15,560 (12,320–17,693)
C-reactive protein (mg/dL)	9.11 (5.02–11.75)
Erythrocyte sedimentation rate (mm/h)	9 (7–17)
NT-pro-BNP (pg/mL)	99.3 (31.3–122.2)
Absolute peripheral eosinophilic count (/μL)	239 (177–603)
Eosinophil % in BAL fluid	45 (37–58)
Chest CT finding	
Diffuse ground glass opacity	20 (91)
Diffuse ground glass opacity with patchy consolidation	2 (9)
Interlobular septal thickening	21 (96)
Pleural effusion	15 (68)
Bedside echocardiography	16 (73)
Oxygen saturation on room air (%)	94 (92–96)
PaO_2_/FiO_2_ ratio	295 (268–314)
Need for oxygen application	21 (96)
ICU admission	8 (36)
Survival	22 (100)

Data are shown as medians (interquartile range) or numbers (%). MMRC, modified medical research council; NT-pro-BNP, N-terminal of the prohormone brain natriuretic peptide; BAL, bronchoalveolar lavage; CT, computed tomography; PaO_2_, partial pressure of arterial oxygen; FiO_2_, fraction of inspired oxygen; ICU, intensive care unit. NT-pro-BNP and PaO_2_/FiO_2_ ratio data were missing in 15 and 3 cases, respectively.

**Fig 1:**
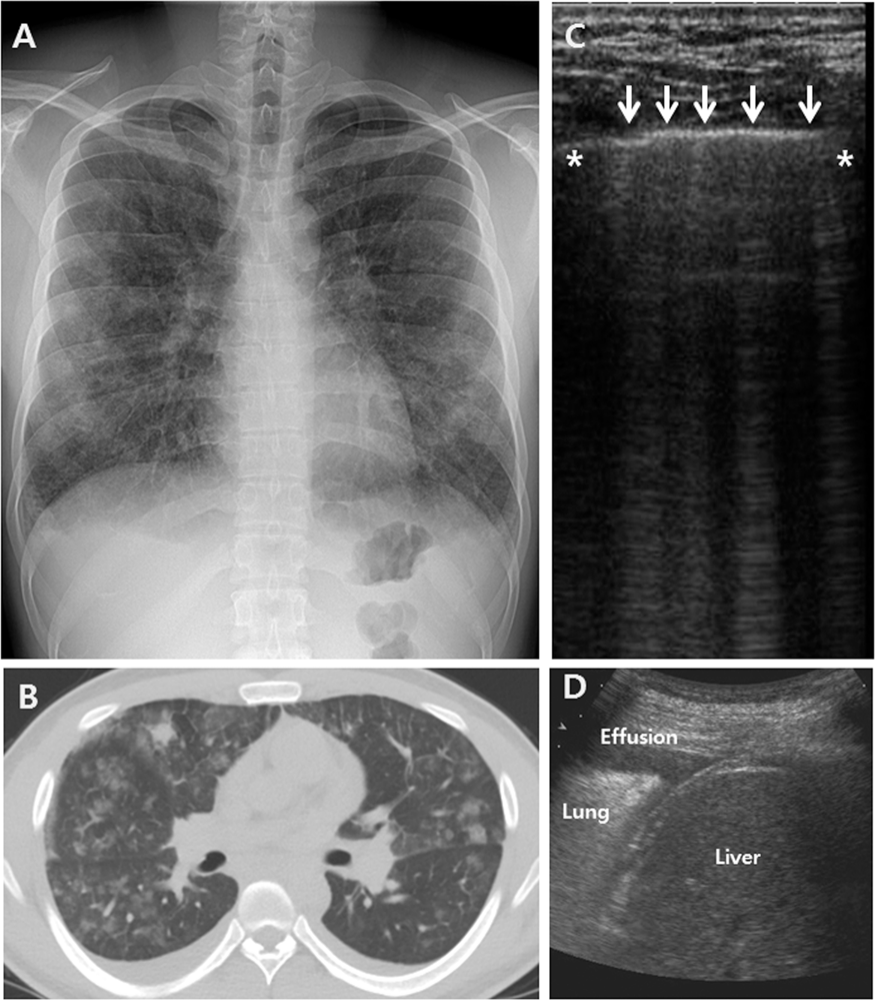
Examples of common thoracic ultrasound (TUS), radiographic, and computed tomography (CT) findings in AEP patients. (A) Chest radiographs revealed bilateral diffuse ground glass opacity (GGO) with reticular opacities and mild blunting of both costophrenic angles. (B) Chest CT images revealed bilateral diffuse GGO with interlobular septal thickening and patchy consolidations bilateral pleural effusion. (C) TUS imaging of the right upper anterior lung zones revealed discrete laser-like vertical hyperechoic reverberation artifacts that arose from the pleural line, multiple B-lines (white arrows) and pleural effusions (D) (asterisk = ribs).

Serum white blood cell counts and C-reactive protein levels were elevated in all patients, with median values of 15,560/μL and 9.11 mg/dL, respectively. Two of seven patients exhibited mildly elevated levels of N-terminal prohormone brain natriuretic peptide (normal: 0–96.2 pg/mL in those aged <45 years). The median value of the initial absolute peripheral eosinophilic count was 239/μL (interquartile range, 177–603/μL, range 132–824/μL), and six of 22 (27%) patients had initial eosinophilia (>500/μL). The median proportion of eosinophils in BAL fluid was 45%. All patients exhibited diffuse ground glass opacity (GGO), with or without patchy consolidation, on chest CT scans. Of all patients, 21 (96%) exhibited interlobular septal thickening, and 15 (68%) had pleural effusion (Fig 1A and 1B). Sixteen (73%) patients underwent bedside echocardiography, and all exhibited preserved heart function.

Median oxygen saturation on room air was 94%, and the median PaO_2_/FiO_2_ ratio was 295. Twenty-one (96%) patients required oxygen delivered via nasal prongs or a facial mask. Eight (36%) patients were admitted to the ICU because of respiratory failure. Of these eight patients, five received high concentration oxygen via a high-flow nasal cannula, and three received non-invasive ventilation. All patients were administered corticosteroid for 2 weeks, and all recovered completely without complications.

### Initial thoracic ultrasound findings

All 22 patients with AEP underwent TUS examinations at admission, and 20 (91%) underwent TUS before diagnostic bronchoscopy, whereas two (9%) underwent TUS immediately after bronchoscopy. The feasibility of all TUS examinations was 100%, and the duration of the TUS examination was <5 min for all patients. The TUS findings according to thoracic area in the patients with AEP are shown in Table 2. All patients exhibited multiple diffuse bilateral B-lines with preserved lung sliding and normal pleural lines (Fig 1 and S1 File). Nine patients exhibited two-sided pleural effusion, four had right-sided effusion, and two had left-sided effusion. Examinations of the left lower anterior lung areas were compromised by cardiac shadows in two patients. Views of the right (*n* = 3), left (*n* = 2), and both (*n* = 2) upper posterior lung areas were limited by the scapula (S1–S4 Figs).

**Table 2:** Thoracic ultrasound findings according to thoracic areas in patients with AEP.

		Right lung			Left lung		
		Posterior area	Lateral area	Anterior area	Anterior area	Lateral area	Posterior area
Upper lung zone	Lung sliding	17/17	22/22	22/22	22/22	22/22	18/18
	A-line	1/17	0/22	0/22	0/22	0/22	3/18
	B-line	17/17	22/22	22/22	22/22	22/22	18/18
	Consolidation	0/17	0/22	0/22	0/22	0/22	0/18
	Effusion	1/17	0/22	0/22	0/22	0/22	0/18
Lower lung zone	Lung sliding	9/22	22/22	22/22	20/20	22/22	11/22
	A-line	0/22	0/22	0/22	0/20	0/22	0/22
	B-line	9/22	22/22	22/22	20/20	22/22	11/22
	Consolidation	0/22	0/22	0/22	0/20	0/22	0/22
	Effusion	13/22	0/22	0/22	0/20	0/22	11/22
	PVL	PAL	AAL	STN	AAL	PAL	PVL

Data are shown as numbers of patients. PVL, paravertebral line; PAL, posterior axillary line; AAL, anterior axillary line; STN, sternum. Examinations of the left lower anterior lung areas were compromised by cardiac shadows in two patients. Views of the right (*n* = 3), left (*n* = 2), and both (*n* = 2) upper posterior lung areas were limited by scapular.

The TUS findings were well explained by those of chest radiography and CT in all study patients. Fig 1 shows an example of common TUS, chest radiographic, and CT findings in the same patient with AEP. The chest radiographs revealed bilateral diffuse GGO with reticular opacities and mild blunting of both costophrenic angles (Fig 1A). The chest CT scans revealed bilateral diffuse GGO with interlobular septal thickening and patchy consolidation. Bilateral pleural effusion was also noted (Fig 1B). TUS imaging of the right upper anterior lung zones revealed discrete laser-like vertical hyperechoic reverberation artifacts that arose from the pleural line, multiple B-lines, [5], and normal pleural lines (Fig 1C). Bilateral pleural effusion was also observed (Fig 1D). Some artifacts were separated by approximately 7 mm on TUS, indicated thickening of the interlobular septa on chest CT, and some other artifacts were separated by 3 mm on TUS, representing GGO lesions on chest CT.

Overall, all 22 patients with AEP exhibited multiple diffuse bilateral B-lines with preserved lung sliding, with (*n* = 15) or without (*n* = 7) pleural effusion. Pleural effusion was common in lower lung areas, and none exhibited a loculated pattern. No definite tissue-like echotexture indicating consolidation was observed on TUS.

### Serial changes in thoracic ultrasound findings during treatment

Serial changes in TUS findings according to clinical, laboratory, and radiological responses during the treatment course are shown in Table 3. Thirteen patients were followed-up for 7 hospital days, at which time they had complete resolution of acute presenting symptoms and all abnormal findings on laboratory, radiologic, and TUS examinations. The remaining nine patients were followed-up for 14 hospital days.

**Table 3:** Serial changes in TUS findings according to clinical, laboratory, and radiological responses.

Characteristics	Day 1	Day 7	Day 14
Disappearance of acute presenting symptoms	NA	22/22	22/22
Inflammatory markers			
Normalization of C-reactive protein	NA	17/22	22/22
Radiographic resolution			
Disappearance of GGO ± consolidation	NA	18/22	22/22
Disappearance of interlobular septal thickening	NA	18/21	21/21
Disappearance of pleural effusion	NA	15/15	15/15
Changes of thoracic ultrasound findings			
Visualization of A-line	NA	19/22	22/22
Disappearance of B-line	NA	13/22	22/22
Disappearance of pleural effusion	NA	15/15	15/15

Data are shown as numbers of patients. GGO, ground glass opacity; NA, not applicable.

**Fig 2:**
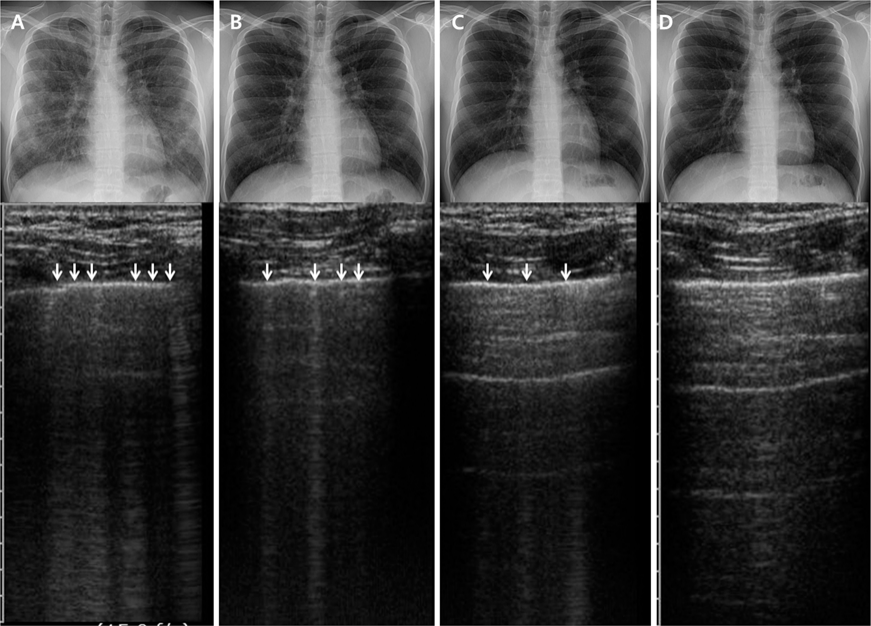
Serial changes in chest radiographic and thoracic ultrasound findings during hospital stays. (A) day 1; (B) day 4; (C) day 7; (D) day 14 (white arrows = B-lines).

All acute presenting symptoms disappeared within 7 days of treatment in all study patients. Serum C-reactive protein levels normalized in 17 patients by day 7 and in all patients by day 14. Eighteen patients exhibited complete resolution of GGO and consolidation as well as interlobular septal thickening on the day 7 radiographic examination. All pleural effusion (*n* = 15) resolved by day 7, and all patients exhibited complete radiographic resolution by day 14. TUS revealed that the number of B-lines had decreased, and the lines became thinner and fainter during the treatment course. A-lines were evident in 19 patients, indicating lung re-aeration, and B-lines had disappeared completely in 13 patients. All pleural effusion (*n* = 15) resolved by day 7. All patients exhibited complete ultrasonic resolution by day 14. Consequentially, both the radiographic and TUS examinations revealed complete resolution of abnormal findings in all patients by day 14. However, some discrepancies were evident between the chest radiographic and TUS findings during follow-up (Fig 2). Chest radiographs taken on day 7 in five patients seemed to show complete resolution, but several abnormal B-lines were evident on a TUS examination performed the same day.

## Discussion

The most important finding in our study was that AEP may commonly present as multiple diffuse bilateral B-lines with preserved lung sliding with or without pleural effusion on a TUS examination, consistent with previous descriptions of alveolar-interstitial syndrome [5, 15]. Lichtenstein *et al*. [5] carefully reported an evaluation of TUS and chest CT findings in 121 patients with pulmonary diseases admitted to the ICU. The abnormal B-lines observed on TUS examinations were mainly due to thickened sub-pleural interlobular septa or GGO; thus, several conditions, including cardiogenic pulmonary edema [16], acute respiratory distress syndrome [17], exacerbation of chronic interstitial lung disease, interstitial fibrosis [15, 18], or specific rare diseases that could involve pulmonary interstitium or alveolar spaces can also present with similar abnormal B-lines on a TUS examination. Fagenholz *et al*. [19] showed that patients with high-altitude pulmonary edema have significantly higher numbers of B-lines on TUS examinations compared with patients with no suspicion of altitude illness. Aghdashi *et al*. [18] also reported that TUS was useful to evaluate the early stages of pulmonary involvement in 31 patients with rheumatoid diseases, including systemic sclerosis, rheumatoid arthritis, Sjögren’s syndrome, and dermatomyositis. However, no data exist on common TUS findings in patients with AEP. Thus, our data may help physicians interpret TUS findings when evaluating patients with acute respiratory symptoms and suggest that AEP may be included in the differential diagnosis, in addition to other conditions that could have interstitial pulmonary involvement, when TUS reveals multiple diffuse bilateral B-lines and preserved lung sliding. However, because TUS findings in patients with AEP may not be disease specific, the diagnosis should be not be made using only TUS findings.

Interestingly, we have shown that TUS may be comparably useful for follow-up and assessing treatment responses in patients with AEP, compared with those of chest radiography, particularly given the high feasibility, low cost, short learning curve, and no ionizing radiation of a TUS examination. As shown in Fig 2, serial changes in the TUS findings, visualization of A-lines, and the disappearance of B-lines were highly correlated with radiographic improvement during the treatment course. Additionally, TUS detected few residual abnormal findings, even when follow-up chest radiography suggested complete resolution. A previous investigation on TUS findings in 300 patients with alveolar-interstitial syndrome [7] also reported that TUS is more sensitive than radiography. In that study, five patients exhibited abnormal TUS findings but normal chest radiographic profiles, and one suffered congestive heart failure. However, few data exist on the clinical applicability or accuracy of TUS when evaluating treatment responses during pulmonary disease follow-up, despite the fact that several studies have suggested the usefulness of TUS [20, 21]. Therefore, our data are of clinical significance and suggest that more aggressive use of TUS may be beneficial when managing patients with AEP.

AEP is an uncommon inflammatory lung disease of unknown etiology. All patients exhibit typical clinical features, including acute-onset respiratory distress with high fever, diffuse pulmonary infiltration, and an increased proportion of eosinophils in BAL fluid, but the detailed pathophysiology remains unknown. Our TUS findings in patients with AEP suggest that interstitial edema caused by inflammatory cell infiltration [22, 23] and capillary leakage may contribute significantly to the development of abnormal radiologic findings and severe hypoxemia in these patients. Additionally, given that no patient exhibited loculated pleural effusion and all exhibited dramatic clinicoradiological responses to corticosteroid therapy alone, our findings indirectly suggest that interstitial edema triggered by inflammation could be a major cause of the observed respiratory distress.

Our study had several limitations. All patients were previously healthy males without underlying lung disease that would have compromised the TUS examination; thus, they were not representative of the general population. Second, our patient number was relatively small. Third, not all patients underwent bedside echocardiography at the time of admission to rule out cardiogenic pulmonary edema. However, given that all patients recovered after corticosteroid treatment alone, and all patients fulfilled the AEP diagnostic criteria, we assumed that none suffered from cardiogenic pulmonary edema. Lastly, because we did not compare TUS findings between AEP and other respiratory diseases that could present as alveolar-interstitial syndrome, an evaluation of specific features for an AEP diagnosis would be limited, thus, further studies are required.

## Conclusions

Our data show the common TUS findings in patients with AEP, including diffuse bilateral B-lines with preserved lung sliding with or without pleural effusion, consistent with alveolar-interstitial syndrome. These results suggest that when TUS findings of alveolar-interstitial syndrome are identified in patients with acute symptoms, AEP may be included in the differential diagnosis in addition to other conditions that show interstitial pulmonary involvement. However, because the TUS findings in patients with AEP may not be disease specific, the diagnosis should not be made only using TUS findings. We also showed that TUS was a useful imaging modality to reliably assess the treatment response and was comparable with chest radiography during the treatment course in patients with AEP.

## Supporting Information

S1 File Video clips of TUS findings in a patient with AEP. (AVI)

S1 Fig TUS findings in a patient 1. (A) Chest radiographs; (B) Chest CT; (C) and (D) TUS imaging of the both upper anterior lung zones; white arrows = B-lines; asterisk = ribs.
(TIF)

S2 Fig TUS findings in a patient 2. (A) Chest radiographs; (B) Chest CT; (C) and (D) TUS imaging of the both upper anterior lung zones; white arrows = B-lines; asterisk = ribs.
(TIF)

S3 Fig TUS findings in a patient 3. (A) Chest radiographs; (B) Chest CT; (C) and (D) TUS imaging of the both upper anterior lung zones; white arrows = B-lines; asterisk = ribs.
(TIF)

S4 Fig TUS findings in a patient 4. (A) Chest radiographs; (B) Chest CT; (C) and (D) TUS imaging of the both upper anterior lung zones; white arrows = B-lines; asterisk = ribs.
(TIF)
