# Supplementary figures and images for: The Utility of Thoracic Ultrasound in Patients with Acute Eosinophilic Pneumonia
Source: PLoS One. 2015 Apr 20;10(4):e0124370. doi: 10.1371/journal.pone.0124370 (PMC4404353; doi:10.1371/journal.pone.0124370)

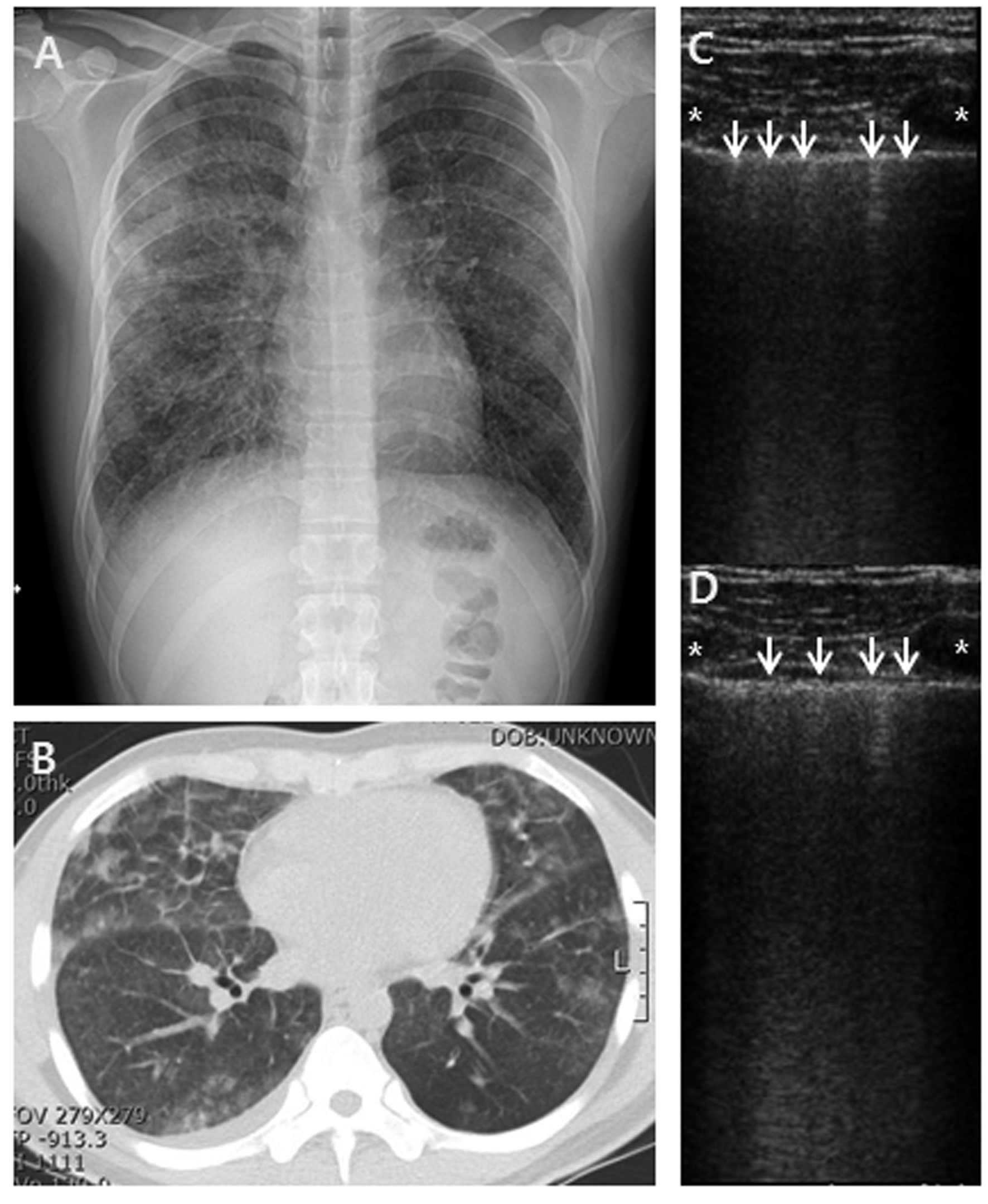

Supplement: S1 Fig — (A) Chest radiographs; (B) Chest CT; (C) and (D) TUS imaging of the both upper anterior lung zones; white arrows = B-lines; asterisk = ribs. (TIF) [file pone.0124370.s002.tif]

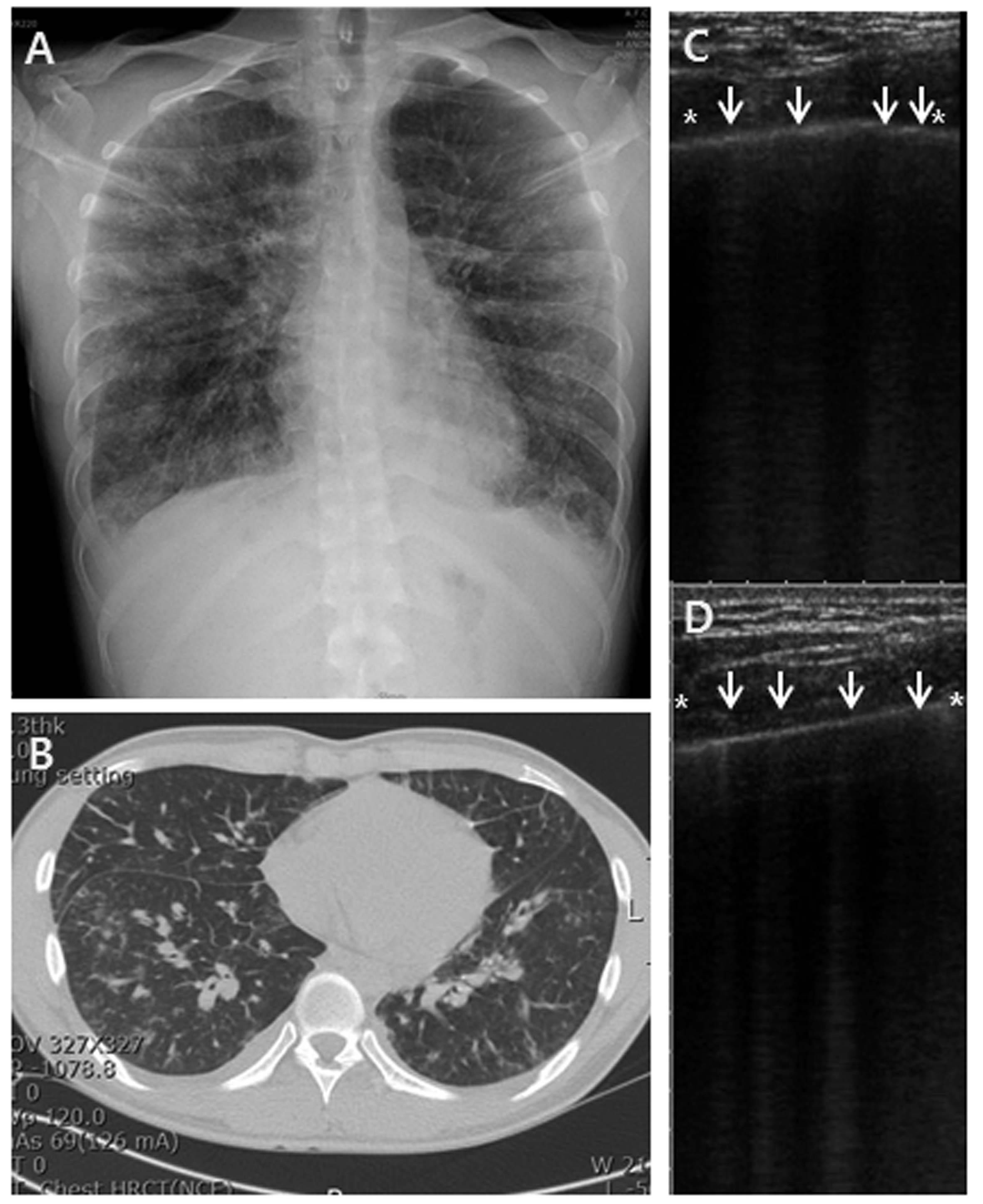

Supplement: S2 Fig — (A) Chest radiographs; (B) Chest CT; (C) and (D) TUS imaging of the both upper anterior lung zones; white arrows = B-lines; asterisk = ribs. (TIF) [file pone.0124370.s003.tif]

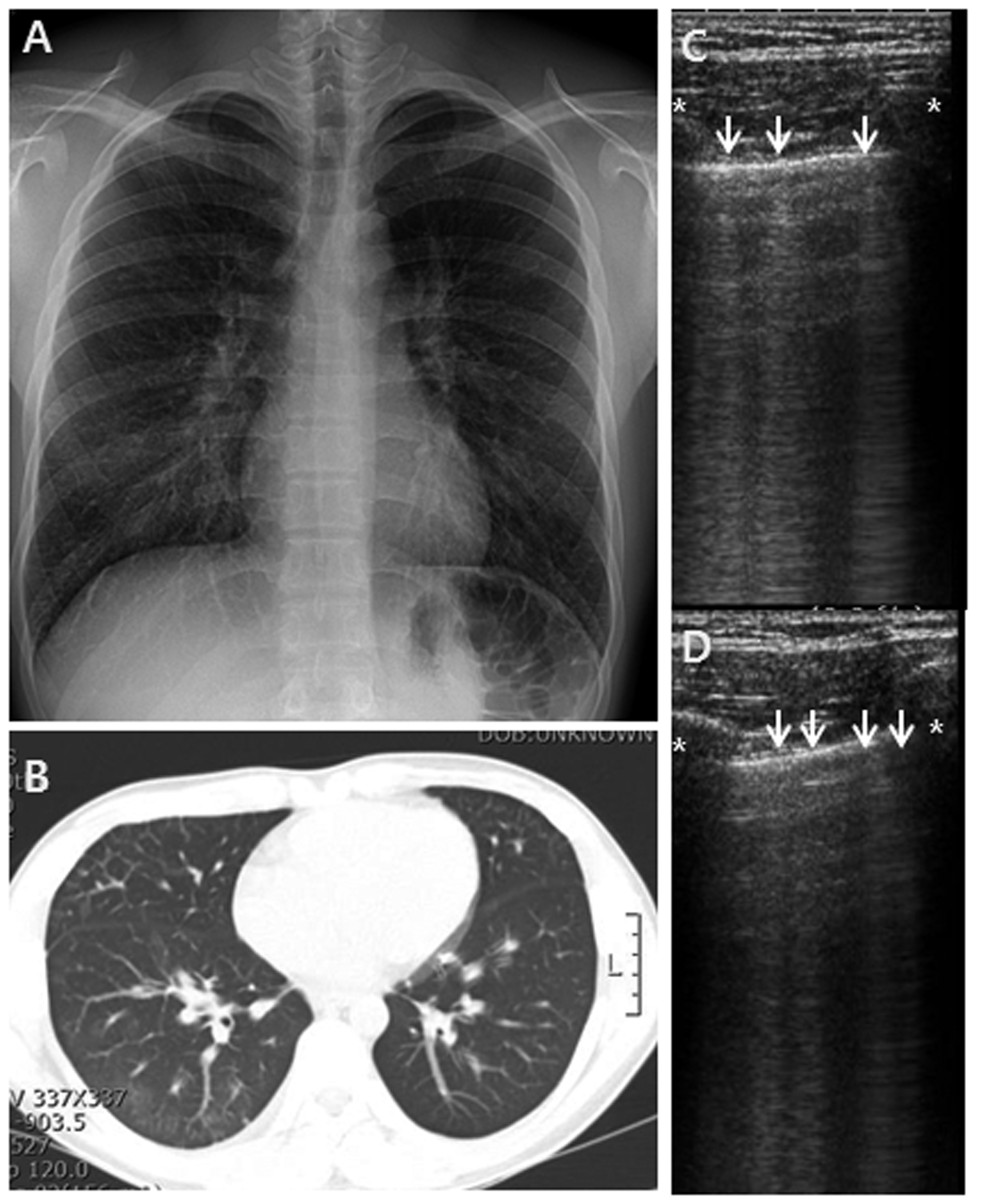

Supplement: S3 Fig — (A) Chest radiographs; (B) Chest CT; (C) and (D) TUS imaging of the both upper anterior lung zones; white arrows = B-lines; asterisk = ribs. (TIF) [file pone.0124370.s004.tif]

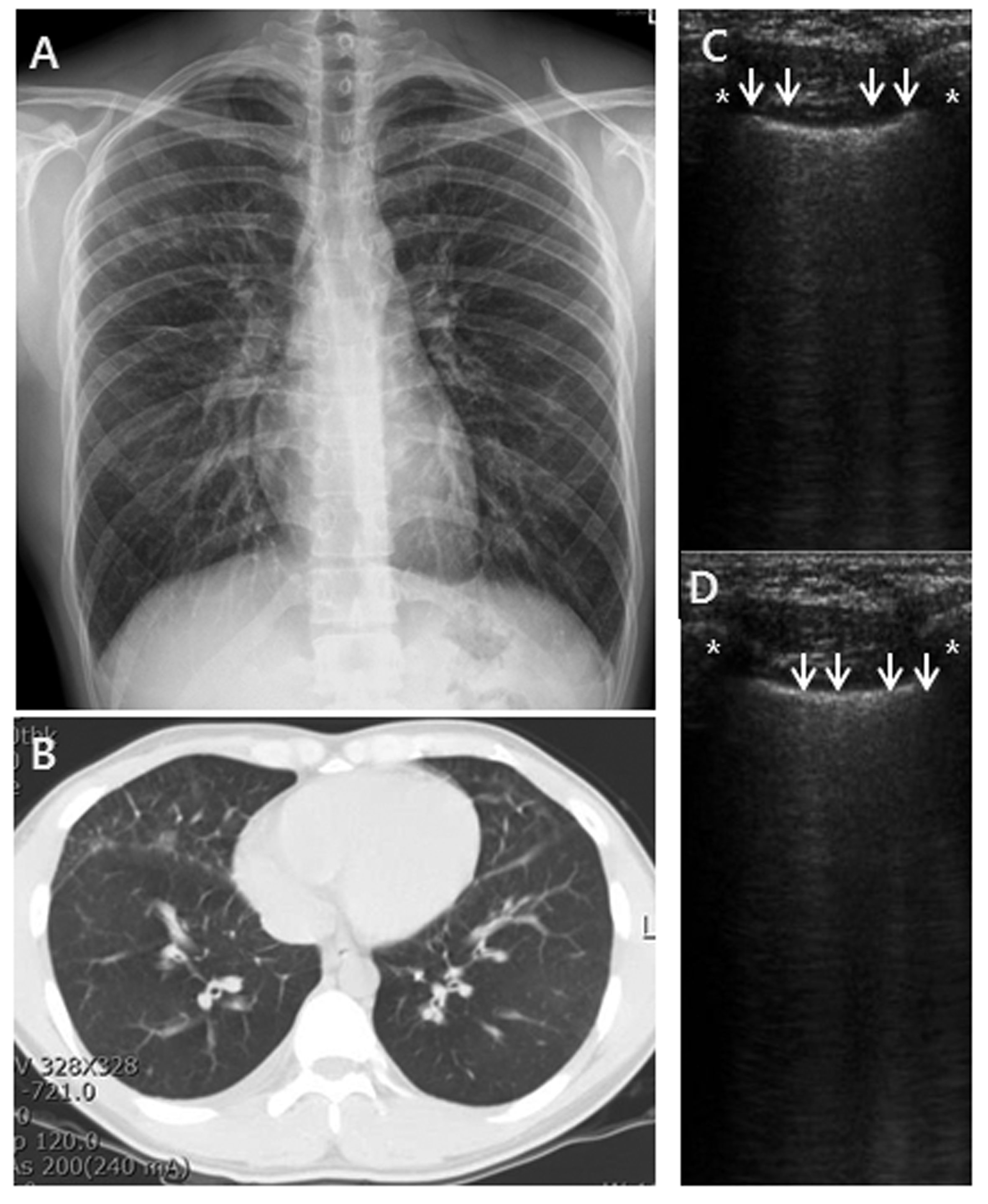

Supplement: S4 Fig — (A) Chest radiographs; (B) Chest CT; (C) and (D) TUS imaging of the both upper anterior lung zones; white arrows = B-lines; asterisk = ribs. (TIF) [file pone.0124370.s005.tif]
